# Oxygen uptake, respiratory exchange ratio, or total distance: a comparison of methods to equalize exercise volume in Wistar rats

**DOI:** 10.1590/1414-431X20165200

**Published:** 2016-07-28

**Authors:** L.S. Paes, J.P. Borges, F.A. Cunha, M.G.C. Souza, F.Z.G.A. Cyrino, D.A. Bottino, E. Bouskela, P. Farinatti

**Affiliations:** 1 Laboratório de Atividade Física e Promoção da Saúde, Instituto de Educação Física e Desportos, Universidade do Estado do Rio de Janeiro, Rio de Janeiro, RJ, Brasil; 2 Laboratório de Pesquisa Clínica e Experimental em Biologia Vascular, Centro Biomédico, Universidade do Estado do Rio de Janeiro, Rio de Janeiro, RJ, Brasil; 3 Programa de Pós-Graduação em Ciências da Atividade Física, Universidade Salgado de Oliveira, Niterói, RJ, Brasil

**Keywords:** Energy expenditure, Aerobic exercise, Exercise testing, Metabolism, Fitness

## Abstract

This study compared strategies to equalize the volume of aerobic exercise performed with different intensities by Wistar rats, based on the distance covered during exercise bouts and energy expenditure (EE, isocaloric sessions) obtained from oxygen uptake (V̇O_2_) or respiratory exchange ratio (RER). Thirty-three male rats (270.5±12.8 g) underwent maximal exercise tests to determine V̇O_2_ reserve (V̇O_2_R), being randomly assigned to three groups: moderate-intensity continuous exercise at speed corresponding to 50% V̇O_2_R (MIC; n=11); high-intensity continuous exercise at 80% V̇O_2_R (HIC; n=11); and high-intensity intermittent exercise (HII; n=11) at 60% V̇O_2_R (3 min) and 80% V̇O_2_R (4 min). Exercise duration was calculated individually to elicit EE of 5 kcal in each session. No difference between groups was found for total running distance (MIC: 801±46, HIC: 734±42, HII: 885±64 m; P=0.13). Total EE measured by RER was systematically underestimated compared to values obtained from V̇O_2_ (HII: 4.5% and MIC: 6.2%, P<0.05). Total EE (calculated from V̇O_2_), and duration of HIC bouts (2.8 kcal and 30.8±2.2 min) were lower (P<0.0001) than in MIC (4.9 kcal and 64.7±1.8 min) and HII (4.7 kcal and 46.9±2.2 min). Predicted and actual values of total V̇O_2_, total EE, and duration of isocaloric sessions were similar in MIC and HII (P>0.05), which were both higher than in HIC (P<0.0001). In conclusion, the time to achieve a given EE in exercise bouts with different intensities did not correspond to the total distance. Therefore, the volume of aerobic exercise in protocols involving Wistar rats should be equalized using EE rather than total covered distance.

## Introduction

Isocaloric exercise bouts aim to equalize the energy expenditure (EE) within aerobic training sessions performed with different intensities (1,2). EE is a surrogate for exercise volume (understood as the interaction between training intensity, duration and frequency) (3,4), and controlling its influence is an important issue in experimental research (2,4). In fact, if the difference between intensities is high enough, exercise sessions performed with a higher intensity might also be considered of greater exercise volume (2,5). This can introduce bias in experiments designed to determine whether a given outcome was produced by exercise intensity or volume. Considering that several benefits of physical training measured by cardiometabolic markers are related to an increase in EE regardless of the intensity of exercise (6–12), exercise bouts should be designed to be isocaloric in studies investigating the specific effects of training intensity or volume (13–15).

In this context, research comparing different exercise intensities in humans adopted rigorous exercise volume equalization (1). This is usually accomplished by measuring the oxygen uptake (V̇O_2_) during exercise to calculate EE. On the other hand, the same concern is not present in most experimental studies with rodents, which have adopted less accurate strategies to isolate the effects of exercise intensity (15–20). Actually, the covered running distance has been primarily used to match the exercise volume across sessions, disregarding differences achieved in EE (16–19,21). Although the assessment of covered distance allows estimation of the performed work (22), it does not assure that EE (and therefore volume) is similar between two exercise sessions or that sessions are isocaloric.

For instance, it is well known that V̇O_2_ kinetics differ according to exercise intensity (i.e., moderate, heavy and severe) and duration (23), which can introduce error into the achieved EE, and consequently in the performed exercise volume. In a practical context, the covered distance can be used to calculate the amount of performed work, but does not provide a precise estimation of total energy production during an exercise bout (24). On the other hand, the EE during exercise has been considered a major determinant of health-related effects (4,8,9) and its correct determination seems to be necessary to optimize the cardio-metabolic impact of physical training programs.

Two major strategies have been usually applied to determine the EE during exercise: a) the quantification of total V̇O_2_ during the exercise bout (4); and b) the calculation of the corresponding energy equivalent per liter of O_2_ consumed (kcal/L) from average respiratory exchange ratio (RER) data (25). Both approaches have limitations that might compromise the accuracy of EE calculation during exercise. For instance, the quantification of total V̇O_2_ does not provide a valid representation of anaerobic EE component (i.e., heavy and severe exercises) and may lead to an underestimation of the caloric expenditure and therefore, of the exercise volume (26). On the other hand, although the RER has been used to calculate EE, there is evidence showing that some metabolic carts are limited in ascertaining short-term changes in this variable, which would compromise the estimation of EE during exercise (27). Therefore, a question arises: which approach (e.g., quantification of total V̇O_2_ or RER) should be applied for a more accurate determination of exercise volume obtained from EE?

To date, no study has examined which method (EE or running distance) would provide a greater accuracy in the equalization of exercise volume and in designing isocaloric exercise bouts for rats. This information would be useful for experiments designed to investigate the specific effects of exercise intensity and volume measuring several outcomes. Therefore, the present study aimed to compare distinct methods for equalizing the volume of aerobic bouts performed with different intensities by Wistar rats. The duration of sessions was calculated based on EE estimated from V̇O_2_ and RER, and the total distance covered during exercise was measured. It is hypothesized that: a) to calculate the exercise duration using EE, equalizing the volume would be more effective than measuring the total distance covered in running bouts; b) to determine the duration of sessions using the EE obtained from V̇O_2_ would be more precise than using RER data.

## Material and Methods

Thirty-three male Wistar rats (*Rattus norvegicus*, 270.5±12.8 g, 12 weeks old, Anilab, Brazil) were kept under a 12-h light-dark cycle in a temperature-controlled environment (22°C) with free access to water and standard rat chow (Nuvital™, Brazil). The experiments were performed according to principles of laboratory animal care (NIH publication No. 86-23, revised 1996) and the protocol was approved by the Ethical Committee of the Universidade do Estado do Rio de Janeiro (#06/2013).

### Study design

After assessing the oxygen uptake at rest (V̇O_2rest_) and during maximal exercise (V̇O_2peak_), the animals were randomly assigned into three exercise groups: a) moderate-intensity continuous exercise (MIC; n=11); b) high-intensity continuous exercise (HIC; n=11), and c) high-intensity interval exercise (HII; n=11). After at least 48 h of the maximal exercise grading test, the rodents underwent the exercise sessions, in a randomized counterbalanced order and within 24-h intervals. The V̇O_2_, EE and total covered distance were measured in each exercise condition.

### Maximal graded exercise test and aerobic sessions

The V̇O_2rest_ and exercise V̇O_2_ were determined by indirect calorimetry via metabolic cart (Oxylet™, Panlab Harvard Apparatus, Spain). The gas analyzer was coupled to a treadmill inside a Plexiglas chamber, connected through a tube to an air pump used to maintain the airflow inside the chamber. The air flow was set at 0.5 L/min during the assessment at rest, and 2.0 L/min during maximal exercise, according to the manufacturer's recommendations. Another tube was connected to the gas analyzer, which continuously measured relative concentrations of oxygen (O_2_) and carbon dioxide (CO_2_) outflowing from the chamber (28). The V̇O_2_ was calculated by a software (Metabolism™, Panlab Harvard Apparatus), using equations described elsewhere (29). Standard conditions of temperature, pressure and humidity were kept in all experiments.

The V̇O_2rest_ was assessed during 30 min and the average of the last 5 min was recorded as final result (30). In order to avoid circadian influence, measurements were taken at the beginning of the animal's dark cycle. Prior to V̇O_2peak_ assessment, in maximal exercise testing, the rats underwent adaptation sessions to the treadmill for 3 days, with speed set at 10 m/min during 10–15 min, as previously described (31,32). Maximal exercise tests were applied 24–48 h following the adaptation sessions. The testing protocol consisted of load increments of 5 m/min every 3 min (28), until the rats were no longer able to run. Exhaustion was determined when animals remained at the end of the metabolic chamber, at the shock grid for 5 s. V̇O_2peak_ corresponded to the highest V̇O_2_ obtained during the maximal exercise test (28).

The target workload during submaximal exercise bouts was calculated using the oxygen uptake reserve (V̇O_2_R) method, as previously described (5): V̇O_2_R = (fraction intensity)×(V̇O_2peak_ − V̇O_2rest_)+V̇O_2rest_. The relative intensity was defined according to each group. Animals assigned to MIC and HIC exercised at speeds corresponding to 50 and 80% of V̇O_2_R, respectively. Exercise sessions for HII consisted of alternate phases corresponding to 60% V̇O_2_R (3 min) and 80% V̇O_2_R (4 min). These exercise protocols were based on previous studies with rats (17,20). Running speeds corresponding to relative intensities were calculated individually, based on V̇O_2_ obtained during the maximal exercise testing (1).

### Calculation of exercise bout duration based on energy expenditure

The duration of isocaloric bouts was calculated as follows: a) first, relative values of V̇O_2_R (mL·kg^-1^·min^-1^) were converted to absolute values (L/min); b) the EE was then calculated (kcal/min) assuming an energy equivalent of 5.0 kcal for each liter of O_2_ consumed (27), and c) the time spent to achieve the targeted total EE (set at 5.0 kcal) was calculated by the ratio between total EE and absolute EE corresponding to V̇O_2_R in each exercise condition (MIC, HIC and HII) (33).

After establishing the speed and duration of the exercise bouts, animals underwent two exercise sessions, during which V̇O_2_ and total covered distance were individually calculated by means of indirect calorimetry system (Panlab Harvard Apparatus). The EE during exercise was determined from the V̇O_2_ (L) assessed during the isocaloric exercise bouts (Table 1). The caloric cost of exercise sessions was also calculated using RER, as previously described (19). In this procedure, the EE per liter of O_2_ consumed (kcal/L) obtained from average RER during the exercise is multiplied by the target V̇O_2_ (L/min), and this result is then multiplied by the bout duration to obtain total EE (kcal). Step-by-step procedures to measure the caloric cost of exercise using RER are shown in Table 2.

**Table:**
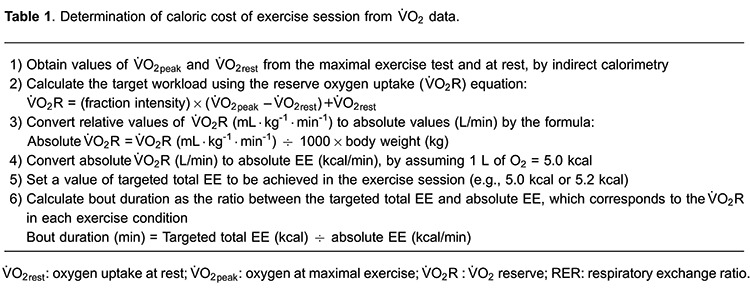

**Table:**
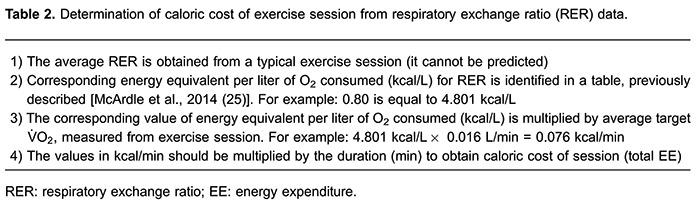

### Statistical analysis

Normal distribution of data was confirmed by the Kolmogorov-Smirnov test and, therefore, results are reported as means±SE. Comparisons for running speed between groups, V̇O_2_, EE, total distance and duration of exercise sessions were made using one-way ANOVA followed by Tukey *post hoc*. Paired *t-*tests were used to test differences between estimated and measured outcomes in each exercise group. In all cases significant level was set at P≤0.05, and calculations were performed using the software GraphPad Prism version 5.00 for Windows (GraphPad™, USA).

## Results

Table 3 exhibits data assessed at rest and during maximal exercise. No significant difference was found between groups for V̇O_2rest_, V̇O_2peak_, and maximal speed achieved in the maximal exercise testing.

**Table:**
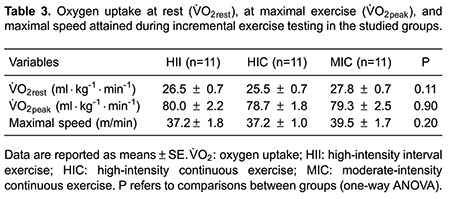

**Figure 1:**
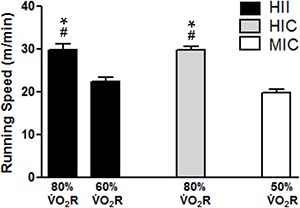
Running speeds (m/min) for each exercise condition (means±SE). V̇O_2_R: reserve oxygen uptake; HII: high-intensity interval exercise; HIC: high-intensity continuous exercise; MIC: moderate-intensity continuous exercise. #P*<*0.0001, compared to HII (60% V̇O_2_R); *P*<*0.0001, compared to MIC (50% V̇O_2_R) (one-way ANOVA followed by Tukey *post hoc*).

**Figure 2:**
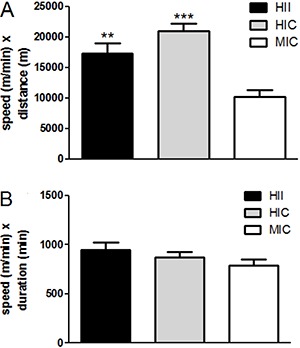
Product between running speed and distance (*A*), and between running speed and duration (*B*). **P<0.01, MIC compared to HII; ***P<0.0001, MIC compared to HIC (one-way ANOVA followed by Tukey *post hoc*).

Running speeds for different conditions and groups are shown in Figure 1. MIC and HIC groups exercised at 19.7±0.8 and 29.8±0.8 m/min, respectively, while running speed in HII ranged between 29.8±1.4 and 22.3±1.0 m/min. Significant differences were found between speeds performed during exercise in 80% V̇O_2_R HII *vs* 60% V̇O_2_R HII (P<0.0001), 80% HII *vs* 50% MIC (P<0.0001), 80% HIC *vs* 60% HII (P<0.0001) and 80% HIC *vs* 50% MIC (P<0.0001). As expected, no significant difference was found between 80% V̇O_2_R HII *vs* 80% HIC (P=0.59). Figure 2 shows data for running speed multiplied by distance (Figure 2A), and running speed multiplied by exercise duration (Figure 2B). These calculations provide results of intensity (running speed) in combination with volume components (distance or duration). Significant differences were found only in regards to running speed *vs* distance data (Figure 2A): the product between speed and distance was significantly lower in MIC than in HII (P*<*0.01) and HIC (P<0.0001).

Table 4 reports results for estimated and measured exercise volume (represented by total EE and V̇O_2_), workload (represented by target EE and V̇O_2_), and duration. The target work V̇O_2_ was higher in HII than MIC (P<0.0001), while no difference was found for estimated total EE either from V̇O_2_ (P=0.66) or RER (P=0.48). On the other hand, total EE (from both V̇O_2_ and RER) was lower in HIC than in both MIC and HII (P*<*0.0001). Only HIC exhibited significant discrepancy between predicted and measured values for total V̇O_2_ (P*<*0.0001), total EE (P*<*0.0001), and duration of exercise bout (P*<*0.0001).

**Table:**
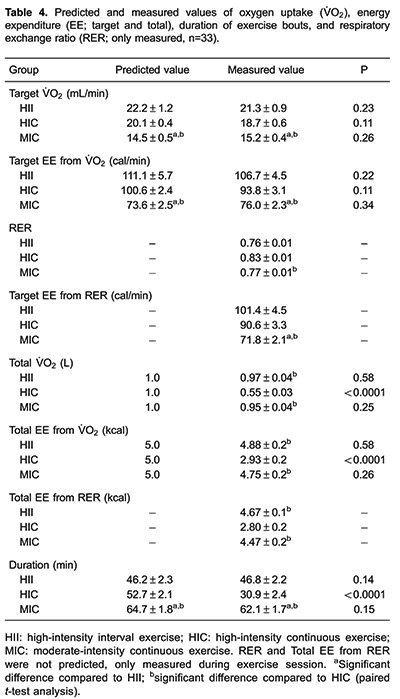

Despite of the disparities between protocols in regards to V̇O_2_ and EE, no significant difference between groups could be detected for the total distance covered in the exercise sessions (MIC: 801±46 m;HIC: 726±45 m; HII: 885±64 m; P=0.13). The EE during exercise calculated from V̇O_2_ was higher than calculated from RER in all groups (P<0.0001). The total EE calculated from RER was underestimated compared to values obtained from V̇O_2_ in HII (4.67±0.1 *vs* 4.88±0.2 kcal; P=0.04) and MIC (4.47±0.2 *vs* 4.75±0.2 kcal; P=0.02), but not in HIC (2.80±0.2 *vs* 2.93±0.2 kcal; P=0.63).

## Discussion

The present study compared different methods for equalizing the exercise volume performed with different intensities by Wistar rats. The main findings were: a) the exercise performed with different intensities elicited similar total distances, but different EE; b) the EE estimated from V̇O_2_ was systematically higher than using RER. Additionally, an original strategy to design isocaloric aerobic exercise sessions for rats has been provided, based on procedures often applied in studies with humans (1,4,5).

Our findings demonstrated that although rats in the three intensity groups had covered similar distances during the exercise bouts, HIC did not reach the predicted total EE. Actually, for a given amount of EE (e.g., isocaloric conditions) the distance covered by the animals in HIC was greater than in MIC and HII. It is worth mentioning that early fatigue was more present in HIC than in the other groups. Apparently, the animals were not able to keep the intensity for the time estimated to achieve the predicted EE. On the other hand, due to the higher speed, this fact did not affect the total distance covered during the exercise. In brief, matching the aerobic bouts using the covered distances in this case would overestimate the EE and therefore the exercise volume. This validates our hypothesis that equalizing the distance during exercise does not necessarily assures that sessions are isocaloric and elicit equal exercise volume, as assumed by prior studies (17,20,21).

The distance covered during running exercise provides only a crude estimate of EE (or performed work) (22). The EE (assessed by V̇O_2_ or RER) reflects more precisely the metabolic demand, being acknowledged as a better marker of exercise volume (4). It has been shown that animals may cover similar distances during equivalent aerobic exercise duration while exhibiting distinct caloric expenditures due to several factors, such as mechanical efficiency or aerobic capacity (34,35). This is not different in humans, since it is well known that total EE and V̇O_2_ are not necessarily the same in subjects that run or walk identical distances (36). Therefore, using covered distance to estimate EE may introduce bias and should be avoided when investigating the specific effects of exercise intensity.

The caloric cost of running in our sample is in agreement with data reported in the classical study by Katch et al. (19) (4.8 *vs* 5.2 kcal/h, respectively). That was probably the first study demonstrating that EE estimated from RER could be used to equalize the exercise volume within animal research models. Interestingly, subsequent investigations comparing different intensities exercise bouts performed by animals did not take those results into consideration (16,17,20,21,37,38). In this sense, the present study adds to the current knowledge by revisiting the pioneer work by Katch et al. (19) and validating the calculation of EE as a preferential strategy to equalize the volume of aerobic exercise bouts.

Furthermore, our findings suggest that estimating the exercise duration based on EE calculated from V̇O_2_ would be more adequate than using RER, in the case of Wistar rats. Prior studies have pointed limitations in regards to the use of RER obtained by open-circuit indirect calorimetry to calculate EE in rodents. Factors such as oscillation in the interconversion of macronutrients or lack of stability in pool sizes of CO_2_ and O_2_ make the interpretation of short-term changes in RER difficult (27). In this sense, transient changes in RER particularly present in exercise performed with high intensity and intermittently could be undetected by this system, because of the delay between collection and analysis of the air collected from the chamber (27). The present results concur with this premise, since the EE estimated from RER was significantly lower than values obtained from V̇O_2_. The RER alone also failed to detect differences in the metabolic demand induced by HII and MIC (RER: 0.77±0.01 *vs* 0.76±0.01, P=0.37, respectively; EE: 71.8±2.1 *vs* 101.4±4.5 cal/min, P=0.0001, respectively). In brief, the calculation of EE from V̇O_2_ instead of RER allowed a more precise estimation of the caloric cost of exercise. This information is especially useful in research settings, since the use of V̇O_2_ avoids the need to submit the rats to extra sessions (two or more), in order to confirm the total EE achieved.

Two methodological approaches have been predominantly applied in the literature to calculate isocaloric exercise sessions (5,33): either using Gross EE or Net EE. Gross EE is defined as the total amount of energy spent during a specific activity, including resting EE; on the other hand, Net EE corresponds to the caloric expenditure specifically induced by the exercise bout, being computed as the difference between Gross EE and resting EE (5,33). Although in humans the use of Net EE is often recommended to avoid overestimation of weight loss induced by exercise programs (5), in the present study we have adopted the Gross EE instead of Net EE. Findings of a pilot study that preceded our experiments (unpublished data) showed that the exercise bout duration was overestimated when using Net EE (109% at 50% V̇O_2_R, and 87% at 80% V̇O_2_R), therefore underestimating the EE for a given workload. Gross EE provided a more acceptable and feasible range of durations for the exercise bouts.

The overestimation of exercise duration with Net EE probably occurred due to the fact that rats exhibit higher metabolic rate than humans at rest (∼26.5 *vs ∼*3.5 mL·kg^-1^·min^-1^, respectively) (26,39) and during exercise (40). Compared to rats, humans have a large amount of fat mass with low contribution to overall metabolism. On the other hand, rats expend calories more easily due to higher metabolic rate of their fat tissue and greater heat loss through body surfaces (27). Thus, unlike humans, to rats exercise represents a large and significant additional caloric cost to maintain their vital processes (26,34). In order to calculate the net EE it is necessary to exclude the values at rest, which represent approximately 35% of maximal EE in rats, resulting in significant overestimation when calculating the duration of exercise bouts. This overestimation of exercise duration due to the use of Net EE resulted in an error of ∼5 kcal added in each session, regardless of the intensity of exercise (i.e., twice the value provided by Gross EE). Hypothetically, such inherent error in the calculations could have a high impact on exercise prescription and expose the animals to unnecessary risk due to prolonged metabolic demand and physiological stress.

Given the lack of studies investigating how to estimate the duration of isocaloric sessions in rats, our data provide useful information demonstrating that Gross EE (converted from target V̇O_2_R), is a valid and reproducible strategy to equalize the volume of aerobic exercise. This method represents a viable alternative to accurately predict individual EE during exercise bouts and equalize the amount of training volume in studies with rodents. In practical terms, this means that additional measurements of V̇O_2_ during the exercise sessions would not be necessary.

In conclusion, our findings indicate that the exercise volume within aerobic exercise bouts in Wistar rats should be equalized by using the time to reach a given amount of EE, rather than the total distance covered during exercise sessions. In order to design isocaloric exercise sessions in rats, procedures similar to those adopted in humans appear to be adequate; that is, after measuring maximal V̇O_2_ and converting a particular relative intensity (% V̇O_2_) into kcal/min, the time to achieve a targeted EE can be estimated using the speed corresponding to that intensity. Further research, however, is warranted to verify the reproducibility and precision of these procedures in different rat strains, as well as to investigate their applicability in studies with other animal models.
